# Design and Analysis of a Variable Inertia Spatial Robotic Tail for Dynamic Stabilization

**DOI:** 10.3390/biomimetics5040055

**Published:** 2020-10-25

**Authors:** Xinran Wang, Hailin Ren, Anil Kumar, Pinhas Ben-Tzvi

**Affiliations:** Mechanical Engineering, Robotics and Mechatronics Lab, Virginia Tech, Blacksburg, VA 24060, USA; wxinran6@vt.edu (X.W.); hailin@vt.edu (H.R.); anilks@vt.edu (A.K.)

**Keywords:** robotic tail, variable inertia, ZMP, dynamic stabilization

## Abstract

This paper presents the design of a four degree-of-freedom (DoF) spatial tail and demonstrates the dynamic stabilization of a bipedal robotic platform through a hardware-in-loop simulation. The proposed tail design features three active revolute joints with an active prismatic joint, the latter of which provides a variable moment of inertia. Real-time experimental results validate the derived mathematical model when compared to simulated reactive moment results, both obtained while executing a pre-determined trajectory. A 4-DoF tail prototype was constructed and the tail dynamics, in terms of reactive force and moments, were validated using a 6-axis load cell. The paper also presents a case study where a zero moment point (ZMP) placement-based trajectory planner, along with a model-based controller, was developed in order for the tail to stabilize a simulated unstable biped robot. The case study also demonstrates the capability of the motion planner and controller in reducing the system’s kinetic energy during periods of instability by maintaining ZMP within the support polygon of the host biped robot. Both experimental and simulation results show an improvement in the tail-generated reactive moments for robot stabilization through the inclusion of prismatic motion while executing complex trajectories.

## 1. Introduction

The tail is one of the most distinctive features visible in most vertebrate animal species, from mammals to fish to reptiles. These animals use their tails to assist locomotion in different forms. For example, kangaroos use tails to balance their body midair while hopping [1], while monkeys utilize their tails for climbing and navigating through tree branches [2]. Tuna exhibit excellent propulsion performance using their tails [3] and lizards have been observed leveraging their tails for pitch control and self-righting mid-air while falling [4,5]. Many research studies have highlighted the importance of the tail as a tool for stabilization, self-righting, and position manipulation [6,7]. This has encouraged research into the study of robotic tail-like appendages on bio-inspired robots for enhanced maneuverability and stabilization.

An upward trend in the exploration of tail applications in bio-inspired robotics has been seen in recent years. Lio et al. demonstrated the use of a single degree-of-freedom (DoF) active tail on a kangaroo robot to compensate for unwanted angular momentum in the pitch axis during the air phase generated by a hopping motion [8]. Patel et al. designed a one-degree-of-freedom tail to assist in the turning of high-speed terrestrial robot [9]. That tail design was later developed into a 2-DoF (pitch and roll) rigid tail, rotating in a conical motion to stabilize the roll motion of a four-wheeled vehicle. The system used inverse dynamics in addition to servomotor constraints and torque input to generate desired trajectories for the tailed, wheeled robot [10]. A tail was also designed for a Two-Wheg Robot to assist it with climbing [11]. Suarez et al. also utilized a small scale dual arm and one degree-of-freedom tail to control an aerial robot for flying and guilding [12]. In a recent study, Heim et al. found that a long and lightweight active tail could be more effective and simplify body-pitch control as compared to other tail models with the same moment of inertia [13,14]. This study also demonstrated that the use of a rigid link with a heavy mass at the end provides a simple and effective way to design robotic tails.

Other researchers incorporate more complex mode shapes in their tail designs in order to generate complex moments in multiple planes. A recent trend in tail design has been the use of cable-driven, segmented structures to change the curvature profile and total mass moment of inertia for such robotic tails [13,14]. Rone et al. [13] demonstrated the use of cable-driven continuum robotic tails to generate torques in the roll, pitch, and yaw directions as well as to change the moment of inertia through bending the tails into different curvatures. Multiple linear actuators were used to drive the cables connected to two segments of tail to generate complex bending modes and torque profiles. A high-fidelity distributed parameter model was then used for dynamic control [15]. Instead of using a DC electric motor, piezo actuators were used in a small-sized robot, an insect-sized (142 mg) aerial robot, to allow for rapid dynamic maneuvers and stabilization [16].

Many biped and quadruped robots have been equipped with tails to assist in the control of body attitude. The under-actuated biped robot, Zappa, walks using the moments generated by its tail, where the tail’s changes in orientation enable motion [17]. The MIT Cheetah is also equipped with a 1-DOF tail to generate moment impulses for mid-air attitude adjustment and disturbance rejection while running at high speed [18].

Rigid link tails provide simple and efficient ways to stabilize the robot but can generate only simple moments while continuum robotic tails provide changeable mass moment of inertia property to generate complex moments for dedicated stabilizations at the cost of dedicated controllers and additional actuators. Building upon these previous lines of inquiry, the goal of the presented research is to design and develop a novel robotic tail platform capable of generating moments in the roll, pitch, and yaw directions, and changing the inertial property of the tail in order to help stabilize and manipulate a biped robot while in motion.

Different from other multi-segment tails that either require a large base actuation unit [19] or strong base link to support each heavy self-actuated link [20], this work pursues a simple tail design and the associated control, and the functionality of changing the tail’s inertial properties as multi-segment robotic tails do. In the proposed design, the moment of inertia with respect to the base of the tail robot was made controllable by enabling the variation of the position of an end effector mass using a prismatic joint. The simple design of the tail also reduces the complexity of the needed real-time control, and part manufacturing and assembly. Prior work seeks to control the mid-air attitude of a quadruped robot or uses the simple assumption that all unwanted angular momentum is dissipated into the ground once contact occurs [9,10,18]. This research explores the effects of a robotic tail as a tool to stabilize and dissipate the excess kinetic energy of a biped robot while in contact with the ground. A controller is designed and validated for the robotic tail in order to control the attitude and stability of a simulated biped robot on the ground with excess kinetic energy in the form of an unexpected impact.

The remainder of this work has been divided into the following sections in order to present the design, modeling, validation, and simulation of the system. Section 2 talks about the mechanical and mechatronic design of this robotic tail. Section 3 discusses the forward kinematic and dynamic modeling of the system. Section 4 presents the control architecture of the tail. Section 5 presents a case study for stabilizing a biped robot using the proposed and Section 6 concludes this paper and discusses future work.

## 2. Mechanical and Mechatronic Design

The proposed robotic tail is a prismatic joint, situated on a spherical joint composed of three independent revolute joints, with a moving mass to affect the tails moment of inertia. This design enables the execution of complex loading profiles without the need for significant actuation in the spherical joint. The reduced actuation requirements allow for lower cost and lower performance actuators to be used in the base without sacrificing overall functionality.

### 2.1. Mechanical Design

**Figure 1:**
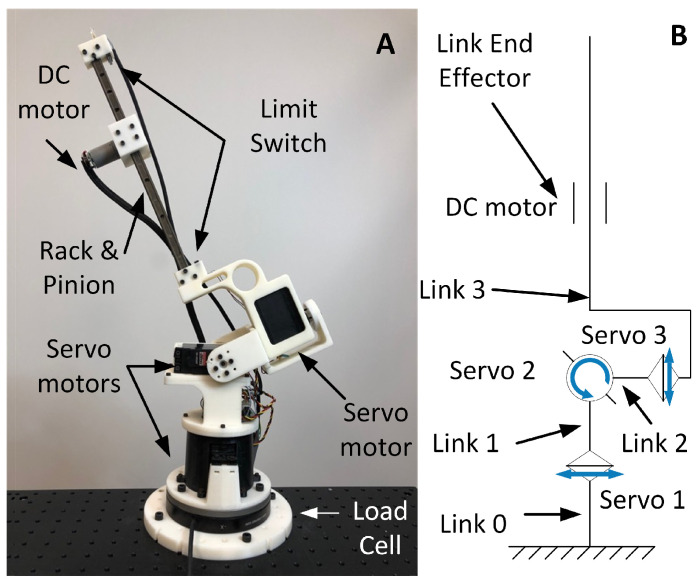
(**A**) The prototype of the robotic tail design. (**B**) Link assignments and details of the robotic tail mechanical components.

This section presents a simple design that enables a 4-DOF rigid tail to achieve both rotation around the *x*, *y*, and *z* axes and translational motion of a moving mass in its local frame. Figure 1A,B show the prototype of the proposed robotic tail and its kinematic diagram, respectively. The three-axis (spherical) rotation is achieved by the three servomotors located at the base, whereas the moving mass is actuated through a custom-made rack-and-pinion linear actuator that can move along the tail main link. Links 1, 2, and 3 are designed to make the rotational axis of the three servomotors intercept at one point, forming a 3-DOF, spherical joint. The rack in Link 3 is made of 1060 aluminum alloy for its high strength yet relatively low weight, while other parts of the tail are made of acrylonitrile butadiene styrene (ABS) material for 3D rapid prototyping. The goal is to reduce the mass of the tail as much as possible with the exception of the moving payload. The payload is composed of a DC motor that actuates the pinion and translates along the Link 3. The motor is equipped with an incremental encoder for position feedback.

### 2.2. Mechatronics Design

**Figure 2:**
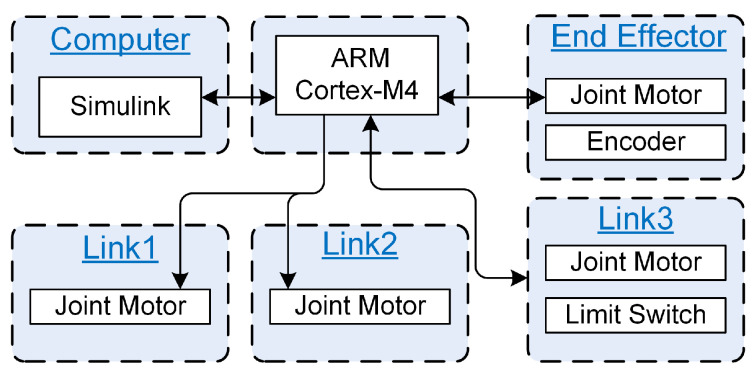
Mechatronic architecture of robotic tail.

To achieve real-time control, a simple mechatronic architecture was developed to control and sense the proposed tail’s pose. As shown in Figure 2, the robotic tail is controlled by an ARM Cortex-M4 microcontroller (located in Link 1). The microcontroller communicates with the host computer over a wired USB connection. The controller receives actuator commands from Simulink™ (running on the host computer) and generates pulse-width modulation (PWM) signals corresponding to the desired position to send to the spherical joint actuators. The controller reads the incremental encoder to record the position of the end effector and uses two limit switches for homing. Position measurements are sent to the host computer (Simulink) for use in the high-level controller.

## 3. System Modeling

### 3.1. Kinematic Modeling

**Figure 3:**
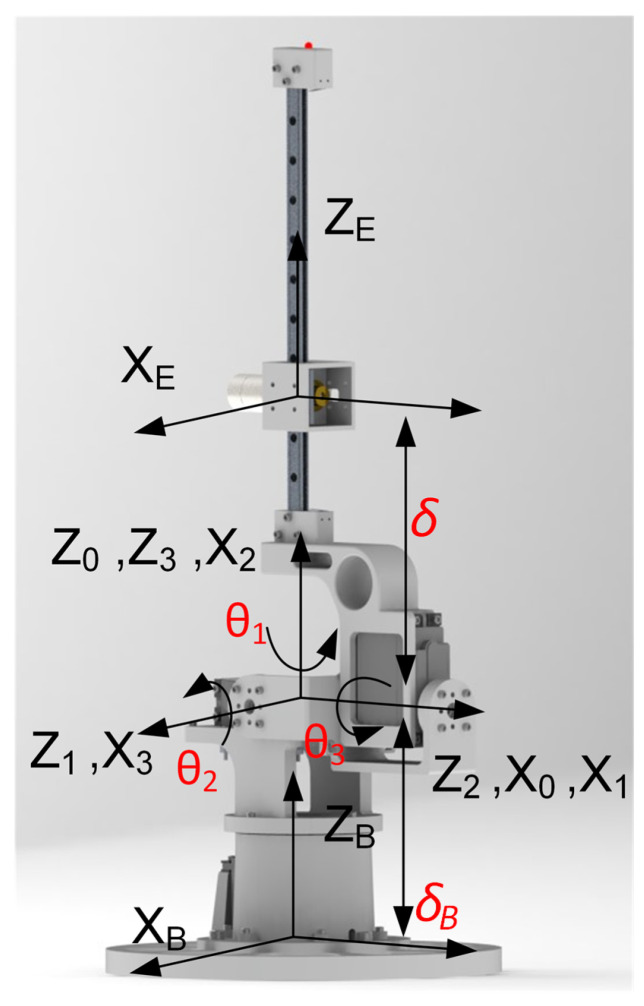
Frame assignments of the proposed robotic tail.

The design of the rigid tail shows that three servomotors rotate the links independently while a DC motor drives the moving mass. The forward kinematics of the proposed tail design were computed using the Denavit–Hartenberg (DH) convention [21]. Figure 3 shows the coordinate frame assignment for each link, where frames 0–2, $\left\{F_{0-2}\right\}$, are attached to Links 1–3 with rotation angles of $\theta_{1-3}$ respectively. The base frame $\left\{F_{B}\right\}$ is attached to the center of the bottom plane of tail robot. The end effector frame $\left\{F_{E}\right\}$ is attached to the moving mass. The scalar value δ defines the translational distance between $X_{E}$ and $Z_{3}$ along $Z_{E}$. The forward kinematics of the robotic tail can then be determined according to DH parameters $\left\{a_{i},\alpha_{i},d_{i},\theta_{i}\right\}$ as listed in Table 1, generated from the frame coordinate assignments. Using the DH convention, a homogenous transformation matrix $A_{j}^{i}$ of any frame $\left\{F_{j}\right\}$ relative to any other frame $\left\{F_{i}\right\}$ can be calculated through the chain multiplication property via an intermediate frame $F_{k}$ using Equation (1) as follows:

(1) $$A_{j}^{i}=A_{k}^{i}A_{j}^{k}|A_{i+1}^{i}=\left(\begin{array}{cc} R_{i+1}^{i}\left(\theta_{i},\alpha_{i}\right) & P_{i+1}^{i}\left(d_{i},a_{i}\right)\\ 0_{1\times 3} & 1 \end{array}\right).$$

Thus, the forward kinematics can be computed by applying the chain multiplication rule in Equation (1) to describe the configuration of the tail through the joint space configuration vector $q={\left[\theta_{1},\theta_{2},\theta_{3},\delta \right]}^{T}$.

### 3.2. Dynamics Modeling

The dynamic model of the proposed tail is built upon the forward kinematics developed in the previous section. Based on the forward kinematics, the linear and angular velocities of each link can be obtained using the following recursive formulation as shown in Equation (2) from the base link to the end effector:

(2) $$\begin{array}{cc} {}^{i}\omega_{i} & =R_{i-1}^{i}\left({}^{i-1}\omega_{i-1}+{}^{i-1}z_{i-1}{\dot{\theta }}_{i}\right)\\ {}^{i}v_{i} & =R_{i-1}^{i}\left({}^{i-1}v_{i-1}+{}^{i-1}z_{i-1}{\dot{d}}_{i}\right)+{}^{i}\omega_{i}\times {}^{i}r_{i}\\ {}^{i}v_{Ci} & =R_{i-1}^{i}\left({}^{i-1}v_{i-1}+{}^{i-1}z_{i-1}{\dot{d}}_{i}\right)+{}^{i}\omega_{i}\left({}^{i}r_{i}+{}^{i}r_{Ci}\right) \end{array}$$

where the terms ${}^{i}\omega_{i}$, ${}^{i}v_{i}$, ${}^{i}v_{Ci}$, ${}^{i}r_{i}$, and ${}^{i}r_{Ci}$, represent the angular velocity of Link *i*, the linear velocity of the origin of frame $\left\{F_{i}\right\}$, the linear velocity of the center of mass (CoM) of Link *i*, the relative position of the origin of frame $\left\{F_{i}\right\}$ with respect to the origin of frame $\left\{F_{i-1}\right\}$, and the position vector of the CoM of Link *i* expressed in $\left\{F_{i}\right\}$, respectively. In addition, the term represents the dimensionless unit vector pointing towards the z-axis of the frame $\left\{F_{i-1}\right\}$ in frame $\left\{F_{i-1}\right\}$ and the control variables ${\dot{\theta }}_{i}$ and ${\dot{d}}_{i}$ represent the rotation velocity of revolute joint *i* and the linear velocity of prismatic joint *i* respectively.

The dynamics of the proposed system are obtained using the Euler–Lagrange method [18]. The Lagrangian for the proposed tail can be expressed as a sum of total kinetic energy $T_{i}$ and potential energy $V_{i}$ of the system using Equation (3):

(3) $$L=\sum_{i=\{B,1,2,3,E\}}\left(T_{i}+V_{i}\right);\left\{\begin{array}{c} T_{i}=\frac{1}{2}m_{i}{\left({}^{i}r_{Ci}\right)}^{⊤}\left({}^{i}r_{Ci}\right)+\frac{1}{2}{\left({}^{i}\omega_{i}\right)}^{⊤}{}^{i}I_{Ci}\left({}^{i}\omega_{i}\right)\\ V_{i}=-m_{i}{}^{0}g^{⊤}{}^{0}p_{Ci}=-m_{i}{}^{0}g^{⊤}\left({}^{0}p_{i}+R_{i}^{0}{}^{i}r_{Ci}\right) \end{array}\right..$$

where, ${}^{0}g$ and ${}^{0}p_{Ci}$ represent the gravity vector and the position vector of the CoM of Link *i* expressed in the frame $\left\{F_{0}\right\}$. With the total energy of the system computed in Equation (3), the joint forces/torques can be computed using the Euler–Lagrange equations of motion using Equation (4) as follows,

(4) $$\tau =M\left(q\right)\ddot{q}+C\left(q,\dot{q}\right)\dot{q}+G\left(q\right)|\tau_{i}=\frac{d}{dt}\left(\frac{\partial L}{\partial \dot{q}}\right)-\frac{\partial L}{\partial q_{i}}.$$

where, $q_{i}$ and ${\dot{q}}_{i}$ represent the displacement and rate of change of displacement of joint *i* and $\tau_{i}$ is the joint torque/force for $i^{th}$ revolute/prismatic joint. In the more general vector formulation of the Euler–Lagrange Equation (4), M(q), $C(q,\dot{q})$, and G(q) represent the mass/inertia matrix of the system, the Coriolis and centrifugal (effect) matrix, and the gravity loading vector, respectively.

The tail motion generates wrench on the tail attachment while the tail base keeps static with respect to its attached body. To estimate the wrench passed by the tail to biped robot, the Euler–Lagrange Method can be extended by adding a virtual 6-DOF joint between the tail base and tail attachment which could rotate about and translate along the *X*, *Y*, and *Z* axis. The base frame $\left\{F_{B}\right\}$ is fixed on the tail base while the virtual frame $\left\{F_{V}\right\}$ is fixed on the biped robot. The attitude and the translation of $\left\{F_{B}\right\}$ with respect to $\left\{F_{V}\right\}$ are described by the Euler angles $\Theta ={\left(\psi ,\theta ,\phi \right)}^{⊤}$ and displacement vector $X={\left(x,y,z\right)}^{⊤}$, respectively. The transformation from $\left\{F_{V}\right\}$ to $\left\{F_{B}\right\}$ is performed by translation along *X*, *Y*, *Z* and then rotation around the *X*, *Y*, *Z* axis of the current frame.

Using these rules, a homogeneous transformation matrix $A_{V}^{B}$, similar to Equation (1), can be made to transform the quantities in $\left\{F_{V}\right\}$ to $\left\{F_{B}\right\}$. For computational advantage, the angular velocity of the base frame $\left\{F_{B}\right\}$, in its own coordinate system is represented as a function of the Euler angle rates $\dot{\Theta }={\left(\dot{\psi },\dot{\theta },\dot{\phi }\right)}^{T}$ in [21] using Equation (5):

(5) $${}^{B}\omega_{B}=\left(\begin{array}{c} \omega_{x}\\ \omega_{y}\\ \omega_{z} \end{array}\right)=\left(\begin{array}{ccc} 1 & 0 & \sin\theta \\ 0 & \cos\psi & -\sin\psi \cos\theta \\ 0 & \sin\psi & \cos\psi \cos\theta \end{array}\right)\left(\begin{array}{c} \dot{\psi }\\ \dot{\theta }\\ \dot{\phi } \end{array}\right).$$

By setting $\left\{F_{V}\right\}$ identical to $\left\{F_{B}\right\}$, $\dot{X}=\dot{\Theta }={\left(0,0,0\right)}^{⊤}$, and assuming infinitesimally small displacement in position, ΔX, and attitude, ΔΘ, ${}^{B}\omega_{B}$ can approximated to $\dot{\Theta }$. This infinitesimal displacement vector can be combined with Equation (4) to estimate the wrench generated by the tail at base.

## 4. Validation of Dynamic Model and Simulation Results

**Figure 4:**
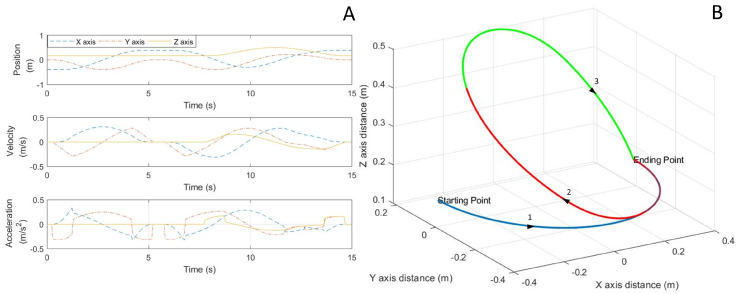
The designed trajectory for the end effector mass. (**A**) Position, velocity, and acceleration of end effector in X, Y, and Z components. (**B**) Positions of the end effector in space.

To validate the dynamic model of the proposed tail simulation, results from the MATLAB™ implementation of the presented model were compared against an (MSC${}^{®}$) ADAMS™ simulation in addition to actual hardware experimentation. To assess the dynamics of the tail, a joint space trajectory, obtained using inverse kinemics, was executed to match the desired end effector trajectory as shown in Figure 4B. In this trajectory, the end effector first traverses through a semi-circle as in trajectory 1 (in the X-Y plane), then upward to the highest point in space as in trajectory 2, followed by a curve to the ending point as in trajectory 3. Figure 4A shows the desired position, velocity and acceleration of X, Y, Z components of the end effector.

In the MATLAB implementation of the proposed dynamic model, the three trajectories of the end effector were simulated and the moment responses at the base of the robotic tail were computed using the Euler–Lagrange method as discussed in Section 3. For the ADAMS study, constraints and joint definitions were added to the imported 3D CAD geometry of the tail and the same end effector trajectories were executed. Similar to the MATLAB study, the moments and forces at the base were measured in ADAMS. Table 2 shows the system used in simulation of the tail dynamics.

For experimental validation of the simulation models, the tail prototype was mounted on a 6-axis load cell capable of measuring forces and torques in real time. The position commands for each joint were sent from MATLAB to the microcontroller using serial communication and the load cell measurements were recorded.

**Figure 5:**
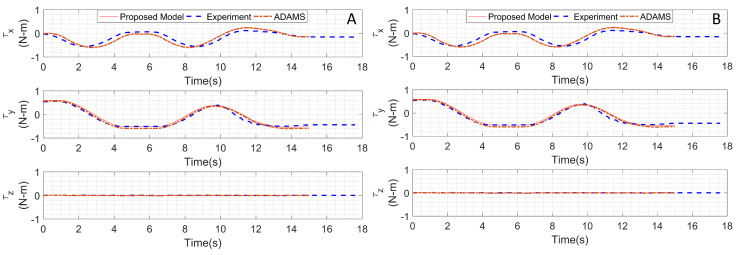
The torques generated at the base when the end effector is at the (**A**) lowest position (**B**) highest position along Link 3.

To study the effect of the end effector position on the wrench exerted by the tail on the base, joint space trajectories were executed where the end effector was positioned at both its lowest (retracted) and highest (extended) possible positions on Link 3. Figure 5A presents the base moments obtained from the proposed mathematical model and ADAMS simulation compared against the experimental measurements collected with the tail in the retracted end effector mode. The magnitude of maximum (absolute) torque estimates from the proposed dynamic model about the *X* and *Y* axis were found to be 0.5706 Nm and 0.5973 Nm, respectively. The maximum moment about the Z-axis was close to zero, as expected due to the near-constant yaw-joint motor speed. The ADAMS simulation results largely corroborate with the proposed model. The maximum moments observed in the experimental data were 0.5592 Nm and 0.5575 Nm along the *X* and *Y* axis, respectively. In comparison to the retracted end-effector mode, following the desired test trajectory in the end-effector extended mode generated higher torques. Figure 5B shows the torque profiles generated from the proposed mathematical model, ADAMS simulation, and experimental data for the end-effector extended mode. The magnitude of the maximum (absolute) torque estimates from the proposed dynamic model about the *X* and *Y* axes were 0.9398 Nm and 0.9567 Nm, respectively, with near zero moments about the Z axis. The maximum moments observed in the experiment about the *X* and *Y* axes were 0.8623 Nm and 0.9280 Nm, and the root mean square (RMS) for the six trajectory between the proposed model and experiment are 0.1099, 0.0742, and 0.0074 for torque in the *x*, *y*, and *z* axis in Figure 5A and 0.1813, 0.129, and 0.0129 for torque in *x*, *y*, and *z* axis in Figure 5B, which are closely matched the simulation results.

The experimental data showed good correlation with that obtained from the simulation models. However, the magnitude of the maximum moment recorded in the experiment is lower than the proposed mathematical model and ADAMS study results. In addition, a de-synchronization in measured torque was observed with respect to the simulation results. Both effects may be attributed to the unmodeled dynamics of the freely hanging wires used to provide power to the DC motor, manufacturing inaccuracies, or component specification deviation. Regardless, the differences are small enough that they are ignored for the remainder of this work.

## 5. Robot Stabilization Using the Robotic Tail

**Figure 6:**
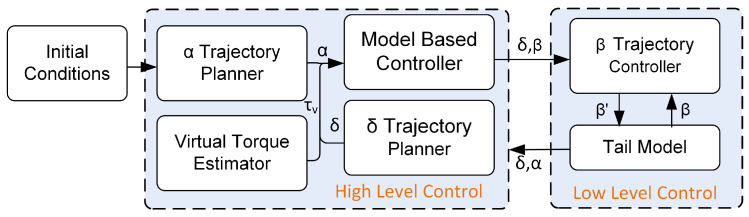
Schematics of the proposed model-based controller.

This paper presents a case study where the applicability of the proposed tail for stabilization is demonstrated on a simulated biped robot after receiving an unexpected angular impulse. A hierarchical controller is developed, as shown in Figure 6.

The high-level controller is composed of the trajectory planner, the zero moment point (ZMP) placement-based virtual torque estimator, and a model-based controller, while the low level controller includes the actuator controller. Based on the robot trajectory, α, obtained from the trajectory planner, the predefined trajectory for δ, and the maximum torque estimated from the ZMP-based virtual torque estimator, the model-based controller can maneuver the tail to generate a counter-moment to bring the ZMP back inside the support polygon and dissipate unwanted energy from the biped robot with the tail actuators. The model-based controller computes the tail, β, and end effector trajectory, which drive the robot tilt angle to follow the desired α and dissipate energy according to the virtual torque estimator. The low-level controller applies proportional integral derivative (PID) control law in order to control the actuators to execute the desired β and δ trajectories.

### 5.1. Biped Robot-Tail System

**Figure 7:**
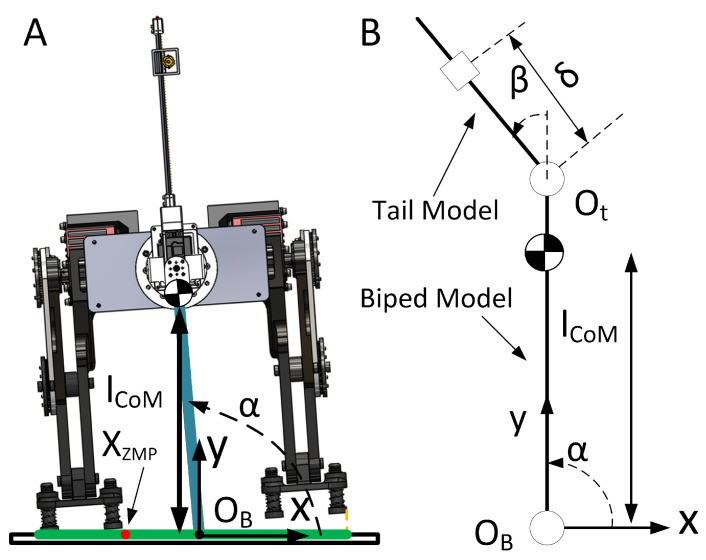
ZMP illustration with simplified mathematical model. (**A**) an illusion of the tail robot attached to a biped robot with a tilt angle α. (**B**) a simplified mathmatical model of the Biped Robot-Tail System with a tilt angle α and actuated tail angle β.

The biped robot is composed of two robotic modular legs [22] with the tail installed horizontally so that the it can rotate about the *z* axis continuously, as shown in Figure 7A. The full system dynamics equation, which was previously simplified, can be expressed using Equation (6) as follows:

(6) $$\frac{dH}{dt}=H_{G}+\tau |H=\sum_{i=\{R,B,1,2,3,E\}}\left(R_{i}^{R}{}^{i}I_{Ci}{}^{i}\omega_{i}+{}^{R}r_{Ci}\times m_{i}{}^{R}v_{Ci}\right),H_{G}=\sum_{i=\{R,B,1,2,3,E\}}{}^{R}r_{Ci}\times m_{i}g^{⊤}$$

where, H is the total angular momentum of the whole system; $H_{G}$ represents the net torque generated by the gravity of all parts; τ is the external torque applied to the entire system; ${}^{i}I_{Ci}$, ${}^{i}\omega_{i}$, ${}^{R}v_{Ci}$, and ${}^{R}r_{Ci}$ represent the moment of inertia, angular rates, linear velocity, and position of the CoM respectively for the $i^{th}$ part in the frame of reference marked by the superscript; sub-index R, represents the biped robot without tail; and sub-indices {B,1,2,3,E} are as defined in Equation (3).

The full dynamic equation is used to simulate the motion of the bipedal robot with a tail, while for the computational convenience a simplified dynamic equation is applied in the inverse model controller, as shown in Figure 7B. The controller models the unstable biped robot as an inverted pendulum with a tilt angle, α, an actuated tail, β, and an end effector mass on the tail at a displacement, δ, with motion that is constrained to the lateral plane. It is also assumed the ground provides enough friction to prevent lateral translation. The rotating joint of the inverted pendulum is located at the center of the support polygon, which is defined by the two robot feet and the direct line connecting them. The rotating active joint of the tail is located at the far end of the inverted pendulum. The simulation parameters used in this case study are listed in Table 3. The mass of end effector is 5 times that of the real tail model.

### 5.2. Trajectory Planner

Rather than mid-air orientation adjustment in which one fixed target tilt angle is pursued, terrestrial vehicles use inverse models to generate actuator trajectories to follow a desired orientation trajectory. Smooth trajectories are always preferred as they avoid abrupt changes in low-level actuation commands and prevent the actuators from saturating. Another concern in trajectory planning is actuation limits, which may cause unpredicted motion and drive the system unstable if the actuators go beyond their limits. A trajectory optimizer is essentially a tradeoff between the trajectory error (difference between desired trajectory and feasible trajectory) and the actuator limit/system health.

While designing a trajectory, the initial and final condition of the system and smoothness of the motion play are most important. The use of higher order polynomials and other continuous functions in modeling the desired trajectory can avoid abrupt changes in actuator commands from the low-level controller. If ${\left\{\alpha \left(t\right)\right\}}_{BC}$ and ${\left\{\alpha \left(t\right)\right\}}_{Feasible}$ are sets for the desired system state trajectories that satisfy the boundary conditions and feasibility limitations, respectively, then the trajectory planner problem converges to finding an optimal $\alpha \left(t\right)\in {\left\{\alpha \left(t\right)\right\}}_{BC}\cap {\left\{\alpha \left(t\right)\right\}}_{Feasible}$. In the presented case, the ${\left\{\alpha \left(t\right)\right\}}_{BC}$ and ${\left\{\alpha \left(t\right)\right\}}_{Feasible}$ are described as follows:

(7) $$\begin{array}{cc} {\left\{\alpha \left(t\right)\right\}}_{BC} & =\{\alpha \left(t\right)|\alpha \left(0\right)=\frac{\pi }{2},∥\alpha \left(t>t_{set}\right)-\frac{\pi }{2}∥<\epsilon ,\dot{\alpha }\left(0\right)={\dot{\alpha }}_{0}\}\\ {\left\{\alpha \left(t\right)\right\}}_{Feasible} & =\left\{\alpha \left(t\right)|M_{inv}\left(\alpha \left(t\right)\right)\in {\left\{\beta \left(t\right)\right\}}_{Feasible}\right\},{\left\{\beta \left(t\right)\right\}}_{Feasible}\}=\left\{\beta \left(t\right)|\beta_{low}\leq \beta \left(t\right)\leq \beta_{up}\right\}. \end{array}$$

where ${\dot{\alpha }}_{0}$ is the initial angular velocity of the biped robot after receiving an unwanted impulse, $M_{inv}\left(\cdot \right)$ defines the inverse model operation, and $t_{set}$ is the desired settling time in which the trajectory converges to within a small acceptable threshold, ϵ, around the final position. In addition, the control variables $\beta_{low}$ and $\beta_{up}$ define the lower and upper limit of the actuator position and velocity for this case study. In order to have a controllable initial and final angular position and velocity, the proposed method chooses an exponentially decaying log function (as shown below) to describe α with design parameters {a,b,c,d} in Equation (8).

(8) $$\alpha \left(t\right)=a\cdot \log\left(1+bt\right)\cdot e^{-ct}+d.$$

where constant *d* determines the desired initial and final position of the biped robot. In our case, the parameter *d* is chosen as π/2 to keep the biped robot vertical. The proposed α model is a high-order differentiable function that guarantees the smoothness of the actuator trajectory. By differentiating the trajectory in Equation (8) with respect to time and solving after equating to zero, the local maximum time, $T_{max}$, can be obtained. The initial condition of angular velocity further constrains the design parameter of trajectory by $b=\dot{\alpha }\left(0\right)/a$, this could be gained by setting t=0 in Equation (9).

(9) $$\dot{\alpha }\left(t\right)=a\cdot b\cdot \frac{1}{1+bt}\cdot e^{-ct}-c\cdot a\cdot log\left(1+bt\right)\cdot e^{-ct}$$

**Figure 8:**
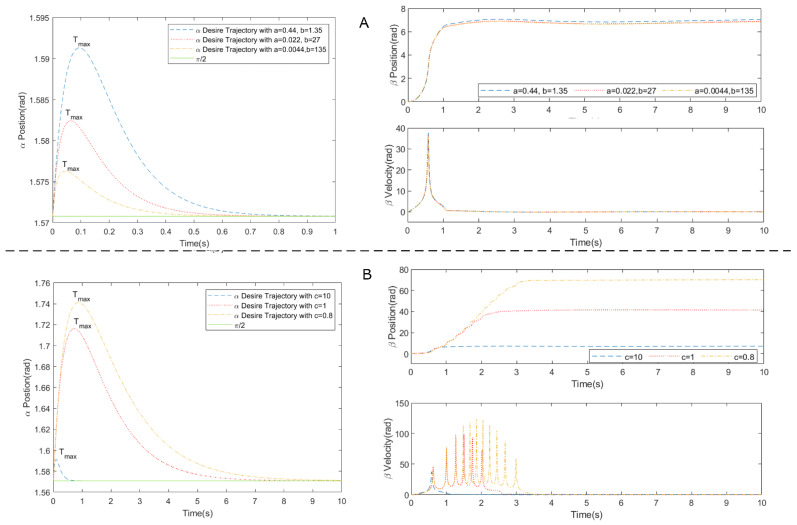
(**A**) α, β and $\dot{\beta }$ trajectories for c=10 and d=π/2 and ${\dot{\alpha }}_{0}=0.587$ rad/s. (**B**) α, β and $\dot{\beta }$ trajectories for a=0.44, b=1.35, d=π/2 and ${\dot{\alpha }}_{0}=0.587$ rad/s with different *c*.

**Figure 9:**
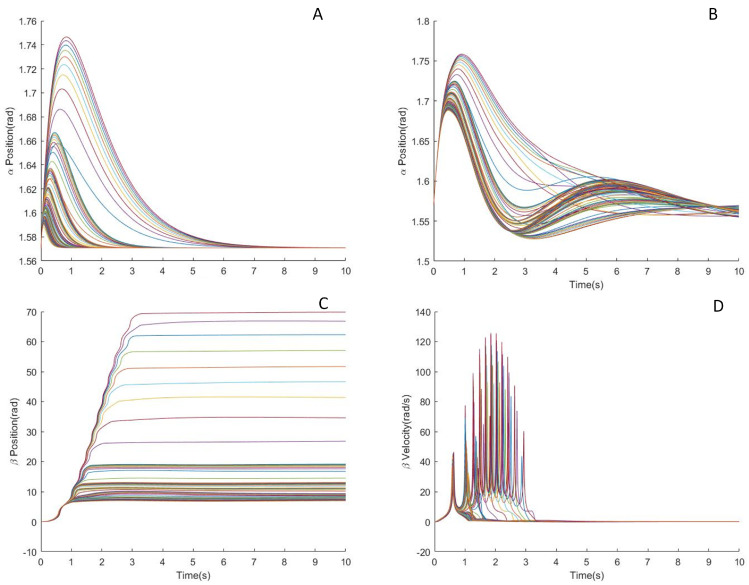
(**A**) Desired trajectory of α. (**B**) Expected trajectory of α obtained from inverse dynamic model. (**C**,**D**) Expected trajectory of β and $\dot{\beta }$ obtained from inverse dynamic model.

Thus, the design parameters of trajectory converge to {b,c}. The constants *b* and *c* determine the position limits of the servomotors and the maximum allowed tilting of the biped robot, as shown in Figure 8. Figure 8A shows the influence of parameter *b* on both trajectory and corresponding actuator trajectory while Figure 8B shows the effect of parameter *c*. The parameters *b* and *c* are obtained by sampling from a predefined set {(b,c)} and carrying out a feasibility study on each sample by applying the inverse dynamic model $M_{inv}\left(\cdot \right)$, described in the following section. Figure 9 shows the real trajectory of both tail actuators and body angles, following desired body angles designed with parameters *b* and *c*. Out of these feasible trajectories, optimal trajectory is then selected considering the overall performance cost. The process can be described in Equation (10) as following,

(10) $$\min_{b,c}\omega_{\alpha }\frac{t_{\alpha_{p}eak|b,c}}{\sum_{b,c}t_{\left(\alpha_{p}eak|b,c\right)}}+\omega_{\beta }\frac{\beta_{\max|b,c}}{\sum_{b,c}\beta_{\max|b,c}}$$

where $\frac{t_{\alpha_{peak|b,c}}}{\sum_{b,c}t_{\left(\alpha_{peak|b,c}\right)}}$ and $\frac{\beta_{\max|b,c}}{\sum_{b,c}\beta_{\max|b,c}}$ are the normalized peak time of α and the normalized maximum value of β, which control the time of the body deviate from the stable stand, and the total rotation of the tail, respectively.

During stabilization, the moving mass simply follows a predefined trajectory, δ, starting at its lowest position to its highest position in order to stabilize by increasing the moment of inertia of the robot. The end effector mass moves at a constant speed in the middle phase of the trajectory with constant acceleration and deceleration in the start and terminal phase. This predetermined trajectory not only minimizes the controller computational, but also reduces mechanical load on the rotary tail actuator that in turn reduces the acceleration and rotation necessary.

### 5.3. Virtual Torque Estimator

Position and orientation control in robots often use zero net angular momentum control in trajectory planning [23,24] whereas terrestrial vehicles more often use moment control for stabilization [25]. The proposed controller utilizes the ZMP to generate a trajectory for energy dissipation and stabilization of the robot-tail system. Multiple researchers in the past have demonstrated the use of ZMP estimates in trajectory planning [17,25]. The ZMP is the point at which the net tipping moment acting on the robot is zero which must be maintained inside the convex hull of the support polygon to prevent toppling of the robot [26]. The green line in Figure 7A on the ground denotes a 1-dimensional projection of the support polygon where the biped robot will stay balanced or recover to stable configuration as long as the ZMP (red dot) is inside the support polygon. In the event of an unexpected impulse, if the ZMP begins to translate outside of the support polygon, the tail can be used to generate counter-moments in order to bring the ZMP back within the support polygon. While the ZMP is inside the support polygon and the tail is still rotating, the virtual torque estimator computes the maximum virtual torque that could be applied to the system to stop the tail from rotating and keep the ZMP inside the support polygon. This motion thus assists the robot in both recovering a stable stance and transferring the excess energy via tail actuators via electromagnetic damping. In this paper, the ZMP based virtual torque estimator computes the current ZMP of the robot (Figure 7) in Equation (11) using [26,27] as follows:

(11) $$X_{ZMP}=l_{CoM}\left(\alpha -\frac{\pi }{2}\right)-\frac{l_{CoM}^{2}\ddot{\alpha }}{g}.$$

Here it is worth noting that as the mass of the robot is ∼19 times larger than the tail and therefore the effect of the motion of the tail on the combined CoM of the whole robot body can be ignored. The CoM of both the biped robot and tail respect to base frame is $l_{CoM}$. After receiving $X_{ZMP}$ from the estimator, the maximum virtual torque that can be applied to the robot while keeping the robot marginally stable can be computed in Equation (12) as follows:

(12) $$\tau_{v}=-\left(l_{CoM}\alpha_{\max}-X_{ZMP}\right)mg\cdot sign\left(H\right).$$

where $\alpha_{\max}$ is the marginally stable value of α and sign(H) is the sign of the angular momentum of the whole system *H*. To avoid discontinuity in the model arising from the sign function in Equation (12), the sign function has been replaced with sigmoidal membership function. The rate of energy that is dissipated can be simplified and expressed using electromagnetic induction principles in Equation (13) as follows:

(13) $$P_{disp}=\frac{dE_{disp}}{dt}=\tau_{v}\dot{\alpha }=P_{act}\left(\dot{\beta }\right)=K_{e}{\dot{\beta }}^{2}$$

$K_{e}$ is the simplified regenerative braking coefficient for DC motors. To avoid discontinuity in the model arising from the sign function in Equation (8), the sign function has been replaced with the sigmoidal membership function.

### 5.4. Model Based Controller

Based on the desired trajectory α and maximum estimated virtual torque, the model-based controller generates actuator trajectory using inverse model dynamics. For computational convenience, the controller uses a simplified model, which treats the tail as a single DoF system and only focuses on the lateral motion as stated in Section 5.1. The simplified model implemented in the controller is stated in Equation (14) as follows:

(14) $$\begin{array}{c} \frac{dH_{sim}}{dt}=G_{sim}+\tau_{v}|G_{sim}={}^{0}r_{C1}\times m_{1}g^{⊤}+{}^{0}r_{C2}\times m_{2}g^{⊤}\\ H_{sim}={}^{0}I_{C1}{}^{0}\omega_{1}+{}^{0}r_{C1}\times m_{1}{}^{0}v_{C1}+R_{2}^{0}{}^{0}I_{C2}{}^{0}\omega_{2}+{}^{0}r_{C2}\times m_{2}{}^{0}v_{C2}\\ =R_{1}^{0}{}^{1}I_{C1}{}^{0}\omega_{1}+{}^{0}r_{C1}\times m_{1}{}^{0}v_{C1}+R_{2}^{0}{}^{0}I_{C2}{}^{0}\omega_{2}+{}^{0}r_{C2}\times m_{2}{}^{0}v_{C2}\\ {}^{0}\omega_{1}=R_{1}^{0}{}^{1}\omega_{1},{}^{0}\omega_{2}=R_{1}^{0}R_{2}^{1}\left({}^{1}\omega_{1}+{}^{1}\omega_{2}\right)\\ {}^{1}\omega_{1}={\left[0,0,\dot{\alpha }\right]}^{⊤},{}^{1}\omega_{2}={\left[0,0,\dot{\beta }\right]}^{⊤} \end{array}$$

where, the quantities ${}^{0}I_{C1}$, ${}^{0}v_{C1}$, ${}^{0}\omega_{1}$, ${}^{0}r_{C1}$, and $m_{1}$ represent the moment of inertia (located at $O_{B}$), linear velocity, angular velocity, center of mass vector, and mass of the leg and tail base (Link 0) with respect to inertial frame $\left\{F_{O}\right\}$ from Figure 7. In addition, the quantities ${}^{0}I_{C2}$, ${}^{0}v_{C2}$, ${}^{0}\omega_{2}$, ${}^{0}r_{C2}$, and $m_{2}$ represent the moment of inertia, linear velocity, angular velocity, center of mass vector, and mass of the tail parts (Links {1,2,3,E}) at their CoM with respect to inertial frame $\left\{F_{O}\right\}$. By inverting the model of the system in Equation (14), the tail control trajectory can be obtained as a continuous function $\ddot{\beta }\left(t\right)=f\left(\alpha ,\dot{\alpha },\ddot{\alpha },\delta ,\dot{\delta },\ddot{\delta },\beta ,\dot{\beta },\tau_{v}\right)$ to be executed by the low-level controller.

### 5.5. Controller Performance

**Figure 10:**
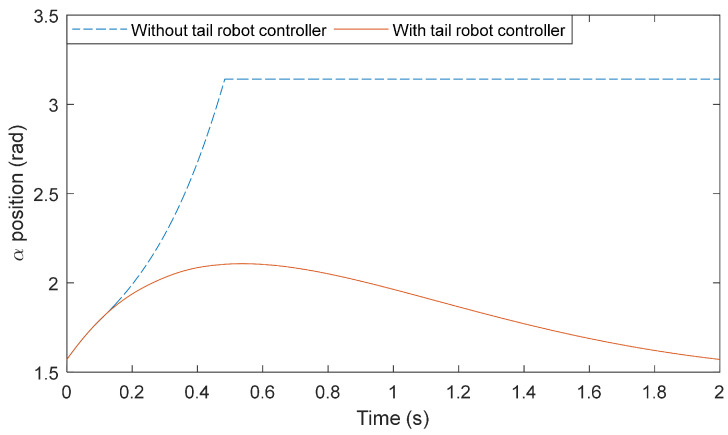
Plot of angle α. Biped robot’s response to external disturbance.

To evaluate the performance of the tail in robot stabilization, the controller was written in MATLAB Simulink™ as applied to the dynamic model of the tail-robot assembly. In the presented case study, the biped robot is simulated to receive an unexpected torque impulse sufficient to drive the ZMP out of the support polygon of the robot. In the absence of the tail controller, the robot becomes unstable and falls to the ground. Figure 10 shows the biped robot trajectory α after receiving an impulse of 10 Nm-s. The simulation study shows that the system recovers to its original orientation through the contribution of tail dynamics.

**Figure 11:**
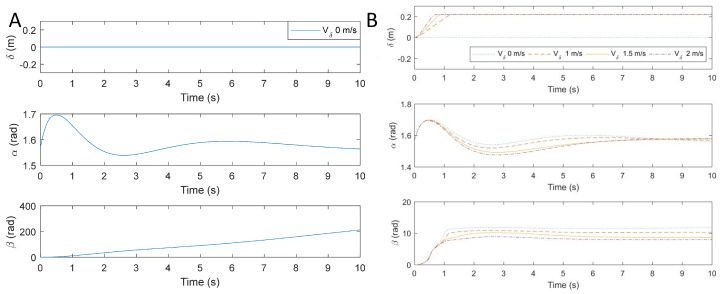
Robot stabilization using tail dynamics: (**A**) without virtual torque estimator. (**B**) With virtual torque estimator in trajectory generation (using end effector linear motion) when subjected to an impulse of 2.5 Nm-s.

**Figure 12:**
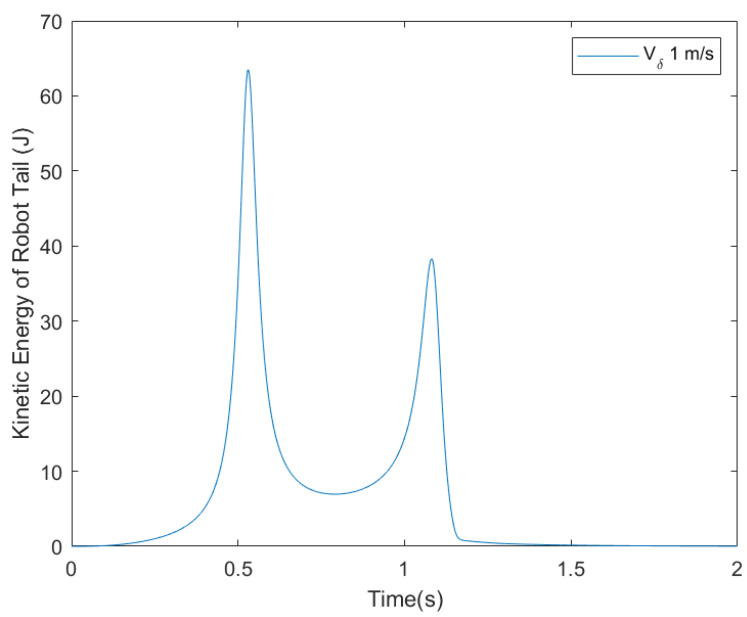
Kinetic energy of the robotic tail vs. time.

The proposed system uses the virtual torque estimator to dissipate the excess kinetic energy. In the absence of the virtual torque estimator, the robotic tail needs to keep rotating in order to balance the unexpected impulse of 2.5 Nm-s (Figure 11A), where angle β keeps increasing in order to stabilize angle α. In reality, the robotic tail has mechanical limits in rotation angles to the design, because the tail cannot balance the unexpected impulse. However, when the virtual torque estimator is added to the robot trajectory planner, the excess energy is imparted to the system due to impulse is dissipated. While adjusting the biped robot’s orientation using the tail, the end effector is moved along Link 3 at different maximum speeds using trajectory shown in Figure 11B. This results in changes to both the α and β angles. It is also worth noting that with the virtual torque estimator in trajectory generation, the tail moves less and stabilizes faster with higher end effector speeds. The simulation results show that the virtual torque estimator can effectively dissipate unwanted energy, hence eliminating the need for the continuous rotation of the tail. Figure 12 shows the energy of the tail during the motion. This energy transfers from the body part to the tail and then transfers back to the body part after the body comes back to the stable zone. Thus, by controlling the position and velocity of the end effector simultaneously, one can limit the tail travel while stabilizing the robot.

## 6. Conclusions and Future Work

This paper presented a novel design of a 4-DoF robotic tail with a demonstrated capability to stabilize a bipedal robot. The incorporation of a prismatic joint in the system helped the tail change its moment of inertia with respect to its base, which could change the magnitude of the moments acting on the tail-robot system all while lowering the actuation requirements for each DoF. The experimental data validated the proposed mathematical model for the tail dynamics, where it delivered up to 0.95 Nm of reactive moment. The case study also validated the controller for a simulated biped robot using the proposed tail as well as demonstrated the capability of the proposed ZMP placement method and momentum-based control in trajectory generation and disturbance rejection. Although the proposed tail robot was well predicted by the model, the power and speed limits of the servomotors restricted its capability to small and lightweight biped robots in the tails current form. In future, the tail will be equipped with more powerful geared brushless direct current electric motor (BLDC) motors to overcome this limitation. In addition, the tail robot will be equipped with a larger mass at the end effector to enable greater control over the moment of inertia of the system. Additional experiments will be performed with a physical bipedal robot, which is already a work in progress as a parallel project [18].

## Figures and Tables

**Table 1:** Denavit–Hartenberg (DH) parameter Table.

*i*	*a*	α	θ	*d*
0	0	0	0	$\delta_{B}$
1	0	90${}^{\circ }$	$\theta_{1}$	0
2	0	90${}^{\circ }$	$\theta_{2}$	0
3	0	90${}^{\circ }$	$\theta_{3}$	0
4	0	0	0	δ

**Table 2:** Tail Parameters.

Parameter	Length (m)	Mass (kg)
Link0	0.094	0.76
Link1	0.073	0.33
Link2	0.139	0.1
Link3	0.416	0.33
Total Mass of Tail	−	1.69
Mass of End Effector	−	0.17

**Table 3:** Biped robot parameters.

Parameter	Unit
Height of Biped Robot	0.482 (m)
$l_{CoM}$	0.382 (m)
Mass of Biped Robot	15.88 (Kg)
Mass of End Effector	0.83 (Kg)
Total Mass of Biped Robot –Tail system	18.3 (Kg)
